# Contribution of Defective PS Recognition and Efferocytosis to Chronic Inflammation and Autoimmunity

**DOI:** 10.3389/fimmu.2014.00566

**Published:** 2014-11-10

**Authors:** Stanley Gititu Kimani, Ke Geng, Canan Kasikara, Sushil Kumar, Ganapathy Sriram, Yi Wu, Raymond B. Birge

**Affiliations:** ^1^Department of Biochemistry and Molecular Biology, Rutgers School of Biomedical and Health Sciences – Cancer Center, Newark, NJ, USA; ^2^Cyrus Tang Hematology Center, Jiangsu Institute of Hematology, First Affiliated Hospital, Soochow University, Suzhou, China; ^3^Sol Sherry Thrombosis Research Center, Temple University School of Medicine, Philadelphia, PA, USA

**Keywords:** phosphatidylserine, apoptotic cells, scramblases, apoptotic versus non-apoptotic PS externalization, autoimmunity

## Abstract

The rapid and efficient clearance of apoptotic cells results in the elimination of auto-antigens and provides a strong anti-inflammatory and immunosuppressive signal to prevent autoimmunity. While professional and non-professional phagocytes utilize a wide array of surface receptors to recognize apoptotic cells, the recognition of phosphatidylserine (PS) on apoptotic cells by PS receptors on phagocytes is the emblematic signal for efferocytosis in metazoans. PS-dependent efferocytosis is associated with the production of anti-inflammatory factors such as IL-10 and TGF-β that function, in part, to maintain tolerance to auto-antigens. In contrast, when apoptotic cells fail to be recognized and processed for degradation, auto-antigens persist, such as self-nucleic acids, which can trigger immune activation leading to autoantibody production and autoimmunity. Despite the fact that genetic mouse models clearly demonstrate that loss of PS receptors can lead to age-dependent auto-immune diseases reminiscent of systemic lupus erythematosus (SLE), the link between PS and defective clearance in chronic inflammation and human autoimmunity is not well delineated. In this perspective, we review emerging questions developing in the field that may be of relevance to SLE and human autoimmunity.

## Introduction

The clearance of apoptotic cells by phagocytic cells (a process now called efferocytosis to distinguish the processing of apoptotic cells from other phagocytic processes) is critically important to maintain homeostasis in multicellular organisms. Efficient efferocytosis not only allows for the removal and degradation of effete and damaged cells, but has an equally important function in the resolution of inflammation by protecting tissue from harmful exposure to the inflammatory and immunogenic contents of dying cells (1–4). There is now considerable genetic evidence supported by mouse knockout studies that failed or delayed efferocytosis results in the release of auto-antigens that can contribute to the etiology of auto-immune diseases such as systemic lupus erythematosus (SLE) (5). In addition, macrophages derived from SLE patients also exhibit defects in efferocytosis (6, 7). Elucidating the genetic basis for defective clearance in relation to human autoimmunity is clearly a topical and important area of research.

Concomitant with caspase activation and cell death, apoptotic cells display a wide array of nascent and modified molecular determinants on their plasma membranes that act as “eat-me” signals for phagocytes. While these determinants result from a combination of re-localized proteins, modified carbohydrates, and from collapse of phospholipid asymmetry at the plasma membrane, the externalization of phosphatidylserine (PS) is arguably the most emblematic event associated with the early phase of apoptotic program (8–10). If apoptotic cells escape immediate clearance, a second wave of late apoptotic cells clearance is mediated by opsonins that includes nuclear materials (11), C1q (12), ficolins (13), and pentraxins (14–16). The late apoptotic cells bound by these opsonins are then recognized and cleared via phagocytic receptors including FcγRIIA, C1q receptor, CR1, CD91, and calreticulin (CRT), helping to avoid inflammation (17, 18). Although our discussion here focuses on cross-interactions between different PS receptors and opsonins, the crosstalk between different recognition systems (such as PS and modified carbohydrates and PS and protein neoepitopes) is likely equally important.

The fact that blockage of PS on the apoptotic cell prevents many of the anti-inflammatory consequences of efferocytosis, combined with observations that knockout of several PS receptors and PS opsonins (soluble factors that link PS on apoptotic cells to receptors) lead to failed efferocytosis, chronic inflammation, and age-dependent autoimmunity (4) has led many investigators to a conceptual framework that externalized PS functions as a dampening platform for negative immune regulation. In this capacity, externalized PS functions both as an “eat-me” signal for efferocytosis, but also as an “inflammo-suppression” signal that promotes tolerance for both immune cells and non-immune bystander cells that come in direct contact with PS externalized membranes (2, 19, 20). Despite convincing evidence as gleaned from knockout studies in mouse, identifying links between defective PS recognition and/or signaling and human autoimmunity has been surprisingly enigmatic (Table 1).

**Table 1:** **Summary of PS receptors and soluble PS binding proteins and their relationship to autoimmunity in mouse and human systems**.

Molecule	Function	Mouse	Human
**PS BRIDGING MOLECULES**			
GAS-6	Bridging molecule between PS and TAM receptor	Deficiency causes platelet dysfunction and protects against thrombosis (21)	Polymorphism positively associated with cutaneous vasculitis in SLE patients (22)
Protein S	Bridging molecule between PS and TAM receptors	Knockout is embryonic lethal (23)	SLE patients have reduced level of circulating protein S (24, 25)
MGF-E8	Bridging molecule between PS and αvβ3/β5 integrins	Deficient mice develops auto-immune disease (26)	Polymorphisms and aberrant splicing reported in some SLE patients (27, 28)
C1q	Acts as PS bridging molecule to SCARF1 and CD91/LRP1. C1q also binds annexin A2, A5, and CRT	Deficiency leads to auto-immune diseases (29)	Ninety percent of C1q-deficient individuals develop SLE (30)
MBL	Bridging molecule between PS and CD91/LRP1	Deficiency leads to defective clearance of apoptotic cells but no auto-immune phenotype (31)	Polymorphisms are SLE risk factors (32, 33)
High molecular weight kininogen	Bridging molecule between PS and uPAR	NR	NR
Thrombospondin	Bridging molecule between PS and CD36	NR	NR
CRT	Binds to PS in a complex with C1q	Knockout is embryonic lethal (34)	NR
**PS RECEPTORS**			
TAM receptors	Indirectly recognize PS via protein S or GAS-6	Tyro-3^KO^/Axl^KO^/Mer^KO^ triple knockout mice develop auto-immune diseases (35). Mer^KO^ single knockout mice develop progressive SLE-like autoimmunity (36)	Polymorphisms in Mer gene associated with multiple sclerosis susceptibility (37). Increased sMertk in advanced atheromata (38) and SLE (39)
Tim-4	Directly recognize PS	Administration of anti-Tim4 mAb into mice caused auto-antibodies production (40)	NR
CD300f	Directly recognize PS	Deficient mice develop a SLE-like disease (41)	NR
SCARF1	Indirectly recognize PS via binding to C1q	Deficient mice developed SLE-like disease (42)	NR
Stabilin-1/2	Directly recognize PS	Deficient mice do not show any SLE-related phenotype (43)	NR
BAI-1	Directly recognize PS	NR	NR
RAGE	Directly recognize PS	Deficiency causes impaired phagocytosis but no SLE-related phenotype (44)	Polymorphism associated with SLE and disease severity in lupus nephritis (45)
CD91/LRP1	Indirectly recognize PS via binding to C1q and/or collectins (MBL, SP-A, SP-D)	Deficient mice are embryonic lethal (46)	SLE patients have significantly increased levels of circulating soluble CD91/LRP1 (47)

*NR, not reported*.

## Mice Lacking PS Receptors are Prone to Lupus-Like Auto-Immune Conditions

Over the past decade, a diverse array of PS receptors and soluble PS bridging proteins that link apoptotic cells to phagocytes have been identified (48–50) (Table 1). Although this suggests significant redundancy at the biochemical level, PS receptors do not appear to act in a compensatory capacity by loss-of-function. For example, on certain genetic backgrounds, single knockouts of Mer (36), Tim-1 (51), Tim-4 (40), SCARF1 (42), and CD300f (41) all have a common phenotype that include defective apoptotic cell clearance, the subsequent production of auto-antibodies, and SLE-like autoimmunity. Similarly, a knockout of MFG-E8 (26), a PS opsonin that bridges apoptotic cells to αvβ5 and αvβ3 integrin, also produces a strong SLE-like phenotype. While in some cases dual targeting of PS receptors can compound phenotypic outcomes [for example Tim-4 and MFG-E8 (52) develop autoimmunity at an earlier age, or triple knockout of TAM (Tyro3, Axl, and Mer) (35) have a more potent onset of disease than Mer alone), collectively these data suggest, at least in the mouse, that PS receptors are not functionally redundant. One possible interpretation is that PS receptors, analogously to the immunological synapse for T cell signaling, comprise a multi-protein signaling receptor complex, perhaps akin to a PS phagocytic synapse, where loss-of-function of any single component disrupts the higher order functional unit (53, 54). Several of the known PS receptors, such as αvβ5 integrin and Mertk, are known to synergize in order to activate intracellular signaling pathways such as Rac1 (55, 56) also supporting the idea of receptor crosstalk. However, while attractive to speculate, such a multi-protein structure (aka, the “engulfosome”) has not been identified at a biochemical level.

Clearly then, an obvious question is whether the aforementioned PS circuitry fails, or is a genetic risk factor for human auto-immune disease such as SLE. Presently, the answer is still not clear, although of the major PS recognition receptors that give rise to autoimmunity in mice (Mer, Tim-1, Tim-4, SCARF1, and CD300f), their involvement in human autoimmunity is not yet obvious from genetic linkage analysis. Although MFG-E8 mutations have been identified in a small subset of lupus patients (28), and a case-control study of MFG-E8 genetic polymorphisms showed some genetic linkage (27), these events appear to be rare. Likewise in the case of TAMs (Mer) and their ligands, it was shown that in SLE patients, TAM levels do not appear to be compromised (57, 58), and in some patients, serum levels of Mer and TAM ligands actually appear to be elevated (59–61).

The recent studies by Ramirez-Ortiz and colleagues, identifying the scavenger receptor SCARF1 (SREC1, CED-1) as a PS receptor that recognizes a PS in the context of complement component C1q (42) might have relevance to human SLE. *In vivo*, SCARF1 (−/−) mice in develop systemic SLE-like disease, including the generation of auto-antibodies and glomerulonephritis that closely mimics human SLE (42). Interestingly, while SCARF1 was shown to bind via PS, apoptotic cells deficient in C1q were notably impaired in their ability to bind to and activate SCARF1, suggesting the C1q acts as a requisite bridging molecule for PS. In addition to SCARF1, C1q also binds to PS-opsonized CRT (62) on the surface of apoptotic cells (a ligand for CD91/LRP1 on the phagocyte), as well as other PS-binding proteins that include Annexin A5 and Annexin A2 (63). Although genetic deficiency of C1q is quite rare (<100 known cases have been reported), over 90% of these individuals develop SLE (30), and monocytes (64, 65) derived from these patients have impaired ability to clear apoptotic cells suggesting a defect in the apoptotic cell clearance machinery. In addition, apoptotic cells derived from SLE patients also show greatly diminished capacity to bind C1q (66) suggesting one or more of the determinants on the apoptotic cell that bind C1q is also deficient in SLE. Although monocytes isolated from SLE patients showed only a modest decrease in CD91/LRP1 levels, patients with rheumatoid arthritis or SLE showed significantly elevated levels of soluble CD91/LRP1 cleaved by ADAM17 in response to inflammation (47). Possibly related, excessive protease cleavage of Mertk from macrophages has also been linked to inefficient clearance in the development of advanced atheromata (38) and SLE (39). Clearly, it will be of interest to ascertain at the genetic level whether loss-of-function mutations occur at CD91/LRP1 or SCARF1 receptor loci that result in risk associations for human auto-immune diseases.

Taken together, while loss-of-function genetic ablation studies in mouse models clearly show a link between systemic autoimmunity and loss-of-function of PS receptors, translating this biology into human SLE pathology still remains somewhat of a mystery. Future studies should address whether PS receptor biology is arranged differently in humans in comparison to mice PS receptors, allowing for more redundancy, or whether defective PS signaling in human is part of a multi-genic signature that acts as a cohort with other risk factors. Another caveat on relying on expression analysis is that many SLE and auto-immune patients are chronically treated with glucocorticoids and steroids, which may affect the levels of PS receptors or PS-opsonins. For example, Lauber and colleagues showed that MFG-E8 is transcriptionally regulated by dexamethasone, a steroid used to treat the chronic inflammation associated with lupus (67). In addition to MFG-E8, the TAM receptors are also subject to acute regulation by glucocorticoids but in a reciprocal fashion; Mer is up-regulated while Axl is down-regulated following dexamethasone treatment (68). This could also induce a feed-forward mechanism, where dexamethasone-induced increase in Mer levels could increase efferocytosis, which itself further increases Mer by the increased uptake of apoptotic cargo. Internalized apoptotic cells increase ingested cholesterol, which can activate LXR and activate the Mer promoter (69, 70). This idea that corticosteroids mediate their effects by manipulating PS biology might be interrogated via the development of more specific therapeutics for SLE.

Another possible reason for the discrepancy between the studies in mice and the observations in human autoimmunity is that defects in PSR signaling (generated in mouse models) may not be manifested as defects in PSRs or PS-opsonins in human autoimmunity but by mutations in genes involved in the mechanisms upstream such as PS externalization or modification. We explore facets of this hypothesis in the following three sections.

## Scramblases, Flippases, and Upstream Mechanisms of PS Exposure

While the past decade has shown great strides in elucidating the repertoire of PS receptors that bind to and rely signals from PS on the apoptotic cell to phagocytic receptors, in recent years, there has also been a much greater appreciation for the genes and regulatory circuits that control PS externalization, including the realization that mutations in these genes can lead to pathologies related to dysfunctional PS biology. Novel scramblases and flippases responsible for PS externalization have been enumerated, opening up the possibility that genes that control externalization, and defects therein, may also contribute to chronic inflammation and autoimmunity.

Similar to other lipids, PS is synthesized in the endoplasmic reticulum and golgi apparatus and then transported to the plasma membrane by carrier proteins. Once PS reaches the plasma membrane, it is actively excluded from the extracellular milieu by several complementary enzymes. These enzymes, in part, maintain membrane asymmetry, with the choline-containing phospholipids; PC and SM predominantly maintained in the outer leaflet, and the amino-phospholipids; PS, PE, and PI predominately on the inner leaflet (71). To maintain PS asymmetry under homeostatic conditions, three main types of enzymes operate at equilibrium, but each can be perturbed during apoptosis and during cell stress. Flippases and Floppases translocate phospholipids from the outer surface to the inner surface and from the inner surface to the outer surface, respectively, and both require ATP for this activity (72). A third, and least understood class of lipid transporters that regulate PS topology are called scramblases, and as their name implies, when activated, collapse membrane asymmetry, and in the context of PS biology, promote the accumulation of PS to the external side of the membrane.

Although phospholipid scramblases do not show selectivity for the phospholipid species or for the direction of movement, the scramblase-mediated exposure of PS has important consequences for several biological events that include coagulation, neurotransmitter release, sperm capacitation, and apoptosis (73). While PS is externalized during both platelet activation and during apoptosis, the recent characterization of two scramblases, Transmembrane protein 16F (TMEM16F) (74, 75) and Xkr8 (76), provide some conceptual relief to this field, highlighting that cells externalize PS through different activation and regulatory mechanisms, but of equal significance, that not all externalized PS has the same biological function.

Transmembrane protein 16F is an eight-transmembrane spanning aminophospholipid scramblase that is critical for the calcium-dependent externalization of PS in activated platelets. In the studies from Nagata and colleagues, these investigators developed a clever FACS sorting approach to characterize a Ba/F3 pro-B cell sub-line that can be trained to respond to sub-threshold concentrations of calcium. After repetitive sorting of PS-positive cells, a Ba/F3 sub-clone that contained a mutated TMEM16F and constitutively scrambled PS was identified (74). Further studies showed that loss of TMEM16F function, either via knockout or through mutation, impairs calcium-dependent PS scramblase activity, and when occurring in platelets, results in their inability to recruit and activate hemostasis factors that include factor V, factor X, and prothrombin to the platelet membrane (75). DNA sequence analysis further showed that Scott syndrome patients, which are characterized by a rare bleeding disorder that have defects on calcium-dependent phospholipid scrambling, carry loss-of-function mutations in both Tmem16 alleles. Functionally, other members of TMEM16 family, including 16C, 16D, 16F, 16G, and 16J are also capable of scrambling PS, but further studies will be required to ascertain whether different family members are specific for different cell types (77).

**Figure 1:**
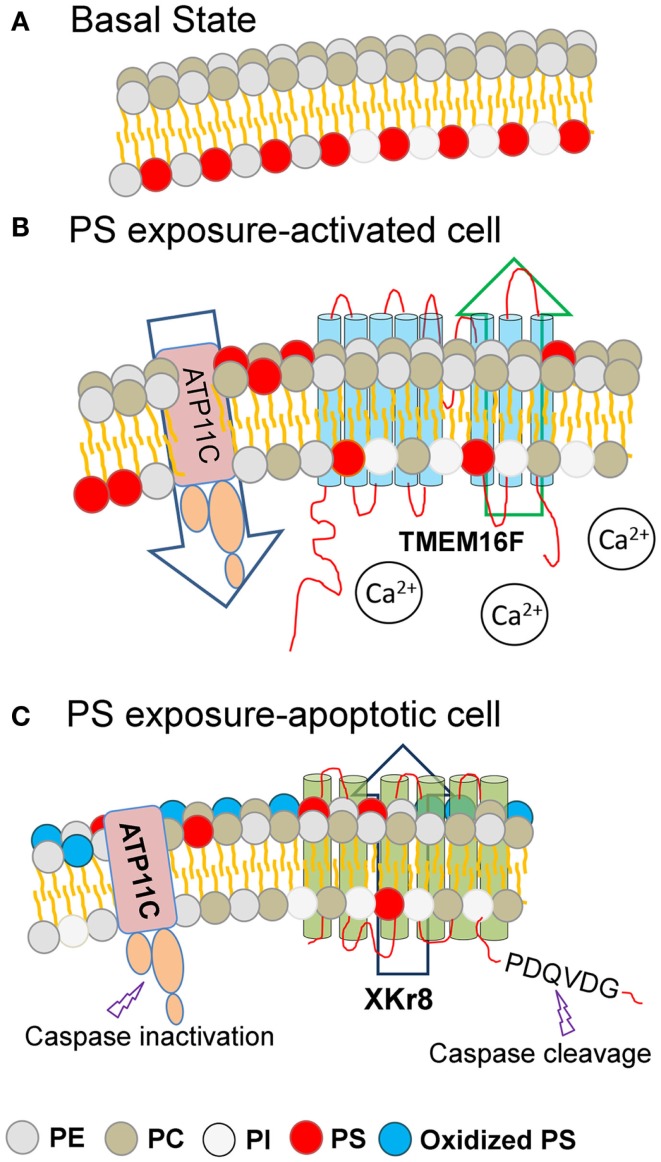
**Different modes of PS externalization by lipid scramblases**. Under basal resting conditions, the amino-phospholipids (PE and PS) are restricted to the inner surface of the plasma membrane **(A)**. During conditions of cell stress or platelet activation **(B)** or during apoptosis **(C)**, membrane asymmetry is lost and PS is externalized to the extrafacial surface (depicted in red). Under basal conditions **(A)**, plasma membrane asymmetry is maintained by the combined activity of cellular flippases and floppases. During cell stress or during platelet activation, intracellular calcium levels rise, resulting in the activation of TMEM16F, and PS exposure to the extracellular leaflet **(B)**. During apoptosis and the activation of caspases, executioner caspases are able to cleave and activate Xkr8, as well as cleave and inactivate ATP11C, resulting in PS exposure to the extracellular leaflet. Since PS externalized PS via TMEM16F and Xkr8/ATP11C are differentially recognized as eat-me signals, it is likely that the density of PS, or the oxidation state of the PS, provide assurance signals for efferocytosis. Key: PE, phosphatidylethanolamine; PC, phosphatidylcholine; PI, phosphatidylinositol; PS, phosphatidylserine; Oxidized PS, oxidized phosphatidylserine.

Notably, the above-mentioned Ca^2+^-stimulated PS externalization induced by TMEM16F is readily reversible upon restoration of Ca^2+^ homeostasis, while the PS externalized during caspase-mediated apoptosis is distinct and separable from TMEM16F, as PS externalization is maintained in apoptotic TMEM16F (−/−) cells treated with Fas-L to induce apoptosis (77). Remarkably, when a mutant TMEM16F was introduced into a mouse lymphoma cell (W3-Ildm) to achieve constitutive PS exposure, these PS-positive tumor cells were not targets of efferocytosis, even by professional DCs, and only became phagocytic competent after treatment with Fas-L to activate caspase 3 (78). These data offer a molecular explanation as to why activated cells, such as during platelet aggregation, T cell activation, and during mast cell degranulation, externalize PS but fail to be engulfed. Conceptually, these data suggest that PS externalization, *per se*, while necessary, is not sufficient to promote clearance (Figure 1).

To identify scramblases associated with apoptosis, Nagata and colleagues used expression cloning to identify scramblases strictly dependent on caspase activity (i.e., inhibited by zFAD-fmk but not dependent on calcium). Based on these screens, a novel scramblase called Xkr8 was identified. Analogous to TMEM16F, over-expression of Xkr8 significantly increased PS exposure, but in stark contrast to TMEM16F, Xkr8 cells that express PS were recognized as an eat-me signal and engulfed. At the molecular level, Xkr8 is cleaved at a DEVD site near its C-termini by caspase 3 and caspase 7 during apoptosis, to activate a PS scramblase activity (76). Xkr8 is a mammalian homolog of the CED8 in *Caenorhabditis elegans* (79) and has an evolutionarily conserved function and is cleaved by CED-3, the homolog of caspase 3, during developmental apoptosis.

Adding complexity to the issue of PS externalization during apoptosis, new studies indicate that a net accumulation of externalized PS is also achieved by a dynamic and systematic interplay between PS scramblases (such as Xkr8) and specific flippases, such as ATP11C (a member of the P4-type ATPase family that redirects PS from the outer membrane back to the inner membrane) (80). Analogous to Xkr8, ATP11C also contains a caspase cleavage site, but when ATP11C is cleaved by active caspases, the Flippase activity is inactivated preventing the return of PS to the inner membrane. Interestingly, when cells express ATP11C with a mutated caspase recognition site, cellular flippase activity remains high, and cells expressing mutant ATP11C do not sustain PS externalization or retain their ability to be engulfed. This presents a highly intricate scenario, whereby caspases can activate Xkr8 and inactivate ATP11C, to increase the steady-state density of externalized PS (Figure 1). In contrast, in the non-apoptotic context, high concentration of calcium activates TMEM16, but does not inactivate ATP11C, possibly explaining the reversibility of TMEM16-mediated PS externalization.

Using an LC MS/MS labeling approach to derivatize primary amines on externalized amino-phospholipids (PE and PS), recent studies by Clark et al. found that different molecular species of amino-phospholipids (according to their fatty acyl composition, saturation, length, and oxidative status) were simultaneously externalized during platelet activation versus apoptosis, and revealed an optimal PE fatty acyl chain length that supported coagulation (81). Similar types of MS-based characterization have been reported to define the molecular species of oxidized PS (oxPS) driven by cytochrome c/H202 (82). These kinds of analyses might be revealing to accesses changes in the PS lipidome in SLE patients, or which species of PS are targets of anti-PS or anti-phospholipid antibodies in SLE. Moreover, the recent development of PS reporter lines, such as the generation of chimeric reporter cells to study the PS-dependent dimerization and activation of TAM receptors (Tyro3-γR1, Axl-γR1, and Mer-γR1 cells) (83), or the use of SCARF1 chimeric receptors to access the contribution of PS to C1q signaling (42), would be very useful to explore the functional analysis for PS receptors and to screen apoptotic cells from different cells undergoing apoptosis (normal versus SLE patients). By expanding this kind of analysis, it might be possible to identify if (and how) PS signaling fails during different externalization itineraries. Together, these studies indicate that not all PS externalization is phenotypically equivalent, and relevant to the thesis developed in this perspective, whether the Xkr8/TMEM16F/ATP11c circuit is compromised or genetically linked to SLE or other human auto-immune disorders is an important and timely question in the field.

## Oxidatively Modified PS may Provide an Assurance Signal for Efferocytosis

The aforementioned discussion between the PS externalization mechanisms of TMEM16F and Xkr8 is instructive, and highlights the fact that PS externalization, *per se*, is not sufficient for efferocytosis. Efferocytosis therefore must require an additional assurance signal, affirming that the cell has passed a caspase-dependent checkpoint and is ready to be engulfed and processed for degradation (84, 85). Although it is likely that other plasma membrane markers act in concert with externalized PS on apoptotic cell, one idea that has gained traction in recent years is that oxPS, generated in a caspase-dependent manner, provides a death-specific marker for PS receptors, marking cells for engulfment (86). oxPS might be expected to change the distribution of PS in the plasma membrane rendering the cell more palatable, or conversely, PS oxidation could serve as a better substrate for PS receptors (i.e., the “altered self” idea) (2).

Although both ideas appear plausible, in support of the latter, it has long been realized that antibodies specific to oxidized phospholipids can block macrophage efferocytosis (87). Moreover, in macrophages, the recognition of apoptotic cells via the scavenger receptor CD36 occurs almost exclusively through interactions with oxPS, and to a lesser extent oxidized PC (oxPC), but not non-oxPS. Interestingly, the specificity of CD36 to oxPS within the apoptotic membranes appears to be mediated by a structurally conserved recognition motif for CD36 that comprises a “sn-2 acyl group with a terminal γ-hydroxy (or oxo)-α, β-unsaturated carbonyl” whereas, the reduction of this acyl chain prevents the oxPS/CD36 receptor activation (88). Other scavenger receptors implicated in apoptotic cell clearance that includes; SRB1, SRA, LOX-1, CD68, and CD14 (2, 89) also appear to selectively recognize the oxidized sn-2 acyl group, suggesting this may be a conserved and universal epitope in the apoptotic program.

In addition to scavenger receptors, recent studies also show that some of the conventional PS-binding proteins and receptors, such as GAS-6 and BAI-1, preferentially interact with oxPS, although in the same study, it was also shown that non-oxPS preferentially bound CXCL16 and Tim-4 (90), suggesting variations on this theme. Although previous studies showed that the peroxidase function of caspase 3 could directly oxidize PS, PS can be oxidized during inflammation as a result of enhanced lipid peroxidation (88). The fact that various oxPS species may alter the repertoire and/or change the affinities of PS toward scavenger receptors and PS receptors provides an impetus to better understand the molecular basis of PS oxidation.

It is also noteworthy that oxysterols and oxPS can also indirectly impinge on efferocytosis. For example, the engulfment of apoptotic cells brings in large amounts of cellular lipids, including the oxidized lipids alluded to above, into the intracellular compartments of the phagocyte. Elegant studies have shown that these internalized lipids can activate PPAR-δ receptors (91) and the nuclear receptor LXR in macrophages (69), to induce engulfment receptors such as Mer and C1q. In mice, genetic ablation of PPAR-δ results in impaired apoptotic cell clearance and SLE-like disease (92), although the significance to human lupus still remains to be determined.

## Lyso-PS, a Unique Form of PS, Binds Distinct Receptors and is Involved in the Clearance of Non-Apoptotic Neutrophils

Finally, in addition to (i) the modes of externalization, (ii) whether PS is covalently oxidized, and (iii) whether a PS receptor is available to bind exposed PS on the surface of the apoptotic cell, under certain circumstances PS can also be hydrolyzed under oxidative conditions by a PS-specific phospholipase (PS-PLA_1_) (93–95) to generate lyso-PS, a deacylated form of PS that serves as an endogenous anti-inflammatory mediator. Although lyso-PS can stimulate efferocytosis under certain conditions (96), this form of PS remarkably also stimulates the uptake of live cells, and has been implicated in the clearance of activated and aged live neutrophils in anticipation for the resolution of inflammation. Despite that PS and lyso-PS have the same anionic head group, lyso-PS does not bind conventional PS receptors such as TAMs and TIMs, but instead interacts with two G-protein coupled receptors, GPR34 and G2A (97), which are linked to novel anti-inflammatory molecules such as PGE2.

## Lessons from Blocking PS in Cancer Models

In recent years, the idea that PS serves as a tolerogenic and global immunosuppressive checkpoint has been therapeutically exploited by the generation of anti-PS antibodies for cancer immunotherapy. These studies show that systemic treatment of Bavituximab (which recognizes a complex of β2-glycoprotein and PS), can activate immune checkpoints, and drive the polarization of macrophages from M2 to M1 and the activation of immature DCs to antigen presenting cells, while decreasing MDSCs and Tregs in tumor-bearing mice (98). As such, this pre-clinical finding has an unanticipated consequence to ask whether blocking PS is sufficient to induce autoimmunity. While the answers are not completely clear, the available pre-clinical and clinical biosafety studies using acute rather than chronic dosing regiments of Bavituximab (anti-PS antibodies), suggest that anti-PS antibodies are well tolerated and do not produce systemic autoimmunity or pulmonary thrombosis (99). Furthermore, vaccinating mice with apoptotic RMA lymphoma cells pre-treated with Annexin-V attenuated the ability of mice to reject a challenge with live RMA lymphoma cells (100). Whether systemic anti-PS treatment exacerbates auto-immune responses in lupus-prone individuals, or in individuals with anti-phospholipid antibody (syndrome), has not been investigated. It will be of interest to identify if patients that develop anti-PS antibodies in SLE might have naturally occurring decreased metastatic burden. Together, these data suggest that blockage of PS, *per se*, may not be causal for the development of lupus, but nonetheless re-activates specific arms of the immune response, which may be fortuitously exploited where immunosuppressive mechanisms operate within the tumor microenvironment. Future studies, in mice, should be aimed to test whether anti-PS antibodies augment lupus-like autoimmunity in genetic strains with a propensity toward disease progression, and conversely whether PS liposomes might also have unexpected therapeutic value. Finally, several enveloped viruses such as Dengue, HIV, and Ebola virus employ apoptotic (PS) mimicry to gain entry to host cells, and blocking PS may also offer therapeutic prospects to block viral entry and immune suppression (101–104).

## Concluding Remarks

While the link between defective efferocytosis and auto-immune disease and advanced atherosclerosis has been made, and validated in experimental animal models, where and when this circuitry fails in human disease has not been firmly established by genetic causation studies. In recent years, new developments have emerged concerning the mechanisms of PS externalization, and the once seemingly simple paradigm that externalized PS provides a signal for efferocytosis and actively drives a resolution in acute inflammation has been refined by the fact that externalized PS can exist in different functional states. A challenging problem in the field will be to decode the different biological fates of externalized PS, and whether its ability to actively transmit signals is compromised in human autoimmunity. Once the specific conditions can be identified, how exactly PS negatively impinges on chronic inflammation can be elucidated further. These data would be helpful to understand what components of the PS pathways fail during chronic inflammation and autoimmunity.

## Conflict of Interest Statement

The authors declare that the research was conducted in the absence of any commercial or financial relationships that could be construed as a potential conflict of interest.
